# Electronic structure and molecular properties of nitisinone and mesotrione in water

**DOI:** 10.1007/s00894-023-05780-5

**Published:** 2023-11-13

**Authors:** Richard Imrich, Juraj Štofko, Roman Boča, Cyril Rajnák

**Affiliations:** 1 Faculty of Health Sciences, University of Ss. Cyril and Methodius, 917 01 Trnava, Slovakia; 2 Faculty of Natural Sciences, University of Ss. Cyril and Methodius, 917 01 Trnava, Slovakia

**Keywords:** Nitisinone, Mesotrione, Molecular properties, Ab initio calculations, Electronic structure

## Abstract

**Context:**

Nitisinone is a medium-sized organic molecule that is used in treating hereditary tyrosinemia type 1 (HT-1). The structurally analogous mesotrione, however, is used as a pesticide/herbicide. What molecular properties are responsible for the similarity/dissimilarity of these molecules is investigated here. The solvent effect reduces the electron affinity to rather negative values and causes the negative electron affinity which manifests itself in a very high positive absolute reduction potential.

**Methods:**

B3LYP method was utilized for a geometry optimization of nitisinone and mesotrione in their neural and ionized (L^0^, L^+^, L^−^) forms of 6 structures. The calculations were conducted in water as a solvent using conductor-like polarizable continuum model (CPCM), nitisinone also *in vacuo*. The complete vibrational analysis at the true energy minimum allows evaluating the thermodynamic functions with focus to the zero-point energy and overall entropic term. The change of the Gibbs energy on reductions and/or oxidation facilitates evaluating the absolute reduction and absolute oxidation potentials. Also, DLPNO-CCSD(T) method that involves the major part of the correlation energy has been applied to nitisinone and mesotrione and their molecular ions.

## Introduction

**Fig. 1:**
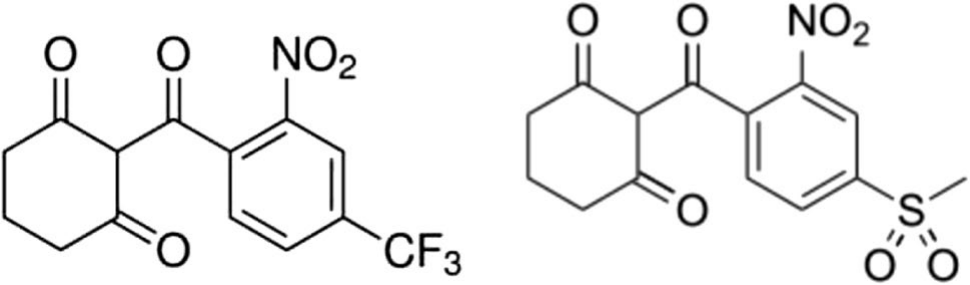
Structural formula of nitisinone (left) and mesotrione (right)

Nitisinone is a medium-sized organic molecule (IUPAC name 2-(2-nitro-4-(trifluoromethyl) benzoylcyclohexane-1,3-dione, C_14_H_10_F_3_NO_5_, 33 atoms) consisting of the substituted aromatic phenyl and non-aromatic cyclohexane rings linked by a ketone bridge (Fig. 1). It contains only two rotatable bonds; thereby, only a limited number of conformers exist. Nitisinone is highly hydrophobic (partition coefficient log*P*_ow_ ~ 2.06, 3.13); it is a white to yellowish-white crystalline powder poorly soluble in water.

Mesotrione (2-(4-(methylsulfonyl)-2-nitrobenzoyl)cycloxexane-1,3-dione, C_14_H_13_NO_7_S, 36 atoms) is an analogous substance to nitisinone, however, having different functional groups attached to the phenyl ring. It contains 3 rotatable bonds and is hydrophobic, log*P*_ow_ = 0.11 (unbuffered water), acidity constant p*K*_a_ = 3.12, solubility in water 1500 mg dm^−3^. It is yellow to tawny solid.

Nitisinone and mesotrione belong to the class of β-triketone herbicides; these are utilized instead of triazine herbicides such as atrazine. Triketones act as inhibitors of the 4-hydroxyphenylpyruvate dioxygenase (HPPD); they, however, increase tyrosine level in plasma. HPPD is an enzyme playing an important role in catabolism of tyrosine.

HPPD, expressed mainly in the liver, converts 4-hydroxyphenyl-pyruvate to homogentisic acid (HGA); it is used in treating hereditary tyrosinemia, type 1 [1]. In 2020, the European Commission has issued the approval of the extended indication for nitisinone to include treatment of adult patients with rare disease alkaptonuria (AKU) based on successful results of the SONIA2 trial [2–6]. Nitisinone is used as a medicament (*Orfadin*) along with restriction of phenylalanine and tyrosine on diet.

Mesotrione is used as a selective herbicide effective mainly to maize. HGA acts as a precursor for synthesis of plastoquinone and α-tocopherol in plants; the inhibition effect to HPPD causes bleaching and death in weed [7]. Mesotrione can cause eye irritation and could contribute to obesity but it is non-toxic by oral consumption. A relationship was found between levels of tyrosine and accumulation of lipid droplets in the non-alcoholic fatty liver disease [8].

The present study focuses to the calculations of the electronic structure of nitisinone and mesotrione and to determine molecular descriptors that cause similarity and/or dissimilarity of these species *in vacuo* and in solvent (water) with focus to redox properties.

## Methods

Ab initio calculations have been utilized in order to get electronic structure and molecular descriptors of nitisinone and mesotrione. Two methods were employed as implemented in the ORCA package [9–11]: DFT-B3LYP, abbr. M1, and DLPNO-CCSD(T), abbr. M2.

B3LYP hybrid variant of density functional theory is effective in the geometry optimization followed by the complete vibrational analysis. As a basis set, def2-TZVPD (valence triple-zeta polarization augmented by diffuse functions) has been applied; it consists of 965 and 998 basis functions with the contraction scheme S-{732,111/511111/211/1}, F-,O-{621,111/4111/111/1}, C-, N-{621,111/411/111/1}, and H-{311/11}; s-, p-, d-, and f-shells are separated by a slash. For open-shell system, the unrestricted variant (UKS) has been applied. The effect of solvation was included by “conductor-like polarizable continuum model” (CPCM) using permittivity *ε*_r_ = 80 for water [12].

In the first step, the structures of nitisinone [13] and mesotrione [14] have been used for the geometry optimization. After the gradient criteria indicated the global energy minimum, a set of molecular properties was evaluated: the energies of HOMO (the highest occupied molecular orbital) and LUMO (the lowest unoccupied molecular orbital), the permanent dipole moment (*p*), isotropic value of the quadrupole moment (*Q*), and the isotropic value of the dipole polarizability (*α*). The vibrational analysis facilitates calculation of the partition function from which the zero-point vibration energy and standard thermodynamic functions (inner energy *U*^ø^, enthalpy *H*^ø^, entropy *S*^ø^, and Gibbs energy *G*^ø^) were evaluated.

Using energies of neutral molecule, molecular cation, and anion, the vertical ionization energy *E*_i_ = *E*^+^  − *E*^0^ and electron affinity *E*_eg_ = *E*^*−*^  − *E*^0^ were calculated in the frozen geometry. These quantities are further processed to get the molecular electronegativity $\chi =({E}_{i}-{E}_{eg})/2$) according to Mulliken, the chemical hardness $\eta =({E}_{i}+{E}_{eg})/2$ according to Pearson, and the electrophilicity index $\omega ={\chi }^{2}/2\eta$ according to Parr [15–17]. The adiabatic variants of the ionization energy and electron affinity were evaluated after the geometry optimization of the respective molecular ions L^+^ and L^−^. The reaction Gibbs energy $\Delta$_r_*G*^ø^ served for the evaluation of the absolute oxidation potential and absolute reduction potential via thermodynamic equation for one-electron process, e.g., *E*_abs_^ø^(L^0^/L^*q*^) [*V*] = –$\Delta$_r_*G*^ø^[J mol^−1^]/*F* with the Faraday constant *F*. The ab initio calculations were conducted also by DLPNO-CCSD(T) method (domain-based local pair natural orbitals–coupled cluster singlets, doublets, and triplets) that account to the major part of the correlation energy. The augmented basis set aug-cc-pVTZ for S-{13,13,13,111/77111/111/11}, F-, O-, N-, C-{88,111/3111/111/11}, and H-{3111/111/11} was applied with 1426 and 1518 basis functions for nitisinone and mesotrione, respectively; the auxiliary basis set was aug-cc-pVTZ/C [18, 19]. The tedious numerical evaluation of the gradient, vibrational displacements, and the polarizability prevents fast geometry optimization and vibrational analysis. Thus, the calculations were conducted in the fixed geometry optimized by the B3LYP method.

## Results and discussion

The set of molecular properties calculated for nitisinone and mesotrione is presented in Table 1. There are reports about three polymorphs of mesotrione confirmed by the X-ray powder diffraction and infrared spectra in the solid state [20–22]. Nitisinone was considered in *in vacuo* as well as in water yielding the following comparisons: (i) HOMO and LUMO energies are the same *in vacuo* and in water [items 1, 2]; (ii) as a solvent effect, the total energies of the neutral molecule and molecular ions are lowered (items 3 through 5); (iii) upon solvation, the ionization energy is lowered and electron affinity adopts more negative values (items 6, 7); (iv) the chemical hardness is reduced as well (item 8); (v) the polar solvent rises the dipole moment substantially (item 10); (vi) the isotropic value of the quadrupole moment is solvent-independent (item 11); (vii) the isotropic dipole polarizability is raised in water (item 12); (viii) the contributions to thermodynamic functions (translational, rotational, and vibrational) are almost insensitive to solvation (items 15–17 and 20–23); (ix) the electronic contributions to thermodynamic functions *U*, *H*, and *G* are influenced by the solvent effect (items 18, 19, and 24); and (x) the enthalpy of hydration is Δ_hyd_*H*^ø^ = *H*^ø^(L^0^_solv_) − *H*^ø^(L^0^_vac_) =  − 15.7 kcal mol^−1^ for nitisinone.

**Table 1:** Molecular properties of nitisinone and mesotrione by DFT-B3LYP^a^

Basis set def2-TZVPD		Nitisinone		Mesotrione
Item		*In vacuo*	In water	In water
1	HOMO	− 171	− 169	− 169
2	LUMO	− 77	− 75	− 75
3	Energy of molecular cation, *E*^+^, frozen str	− 796,531.04	− 796,591.65	− 953,986.85
4	Energy in optimized geometry, *E*^0^	− 796,743.88	− 796,759.13	− 954,154.48
5	Energy of molecular anion, *E*^*−*^, frozen str	− 796,780.67	− 796,836.29	− 954,232.35
6	Ionization energy, *E*_i_ = *E*^+^ − *E*^0^, vertical	213	167	168
7	Electron affinity, *E*_eg_ = *E*^−^ − *E*^0^, vertical	− 37	− 77	− 78
8	Mulliken electronegat., $\chi$_M_ = (*E*_i_ − *E*_eg_)/2	125	122	123
9	Pearson hardness, *η*_P_ = (*E*_i_ + *E*_eg_)/2	88	45	45
10	Dipole moment *p*/debye	2.620	7.345	8.868
11	Quadrupole moment *Q*/e*a*_0_^2^	− 100.1	− 100.4	− 110.2
12	Dipole polarizability *α*/*a*_0_^3^	192.5	270.4	309.5
13	Solvated surface area *S*/*a*_0_^2^	-	1146	1222
14	Solvated volume *V*/*a*_0_^3^	-	2242	2399
15	*E*_vib_(ZPE) − zero point energy	143.12	142.69	163.49
16	Overall *E*_vib_(*T*^ø^) contribution	153.87	153.42	175.08
17	*E*_rot_ = *E*_trs_	0.89	0.89	0.89
18	Inner energy *U*^ø^	− 796,588.25	− 796,603.93	− 953,977.63
19	Enthalpy *H*^ø^	− 796,587.65	− 796,603.34	− 953,977.03
20	*S*_vib_*·T*^ø^ contribution	19.55	19.40	21.06
21	*S*_rot_*·T*^ø^ contribution	10.39	10.38	10.43
22	*S*_trs_*·T*^ø^ contribution	12.90	12.90	12.93
23	Total entropic term *S·T*^ø^	42.83	42.68	44.42
24	Gibbs energy *G*^ø^ in optimized str of L^0^	− 796,630.49	− 796,646.02	− 954,021.46

^a^Energies in units of kcal mol^−1^, 1 hartree = 627.503 kcal mol^−1^; 1 kcal mol^−1^ = 4.184 kJ mol^−1^; 1 eV = 23.0609 kcal mol^−1^; *T*^ø^ = 298.15 K; angstrom Å = 10^−10^ m; bohr* a*_0_ = 5.292 × 10^−11^ m; debye* D* = 3.336 × 10^−30^ C m; *α*(*a*_0_^3^) = 0.1482 × *α*(Å^3^). Abbr. *str* structure

Table 2 brings electronic energies and standard Gibbs energies of nitisinone and mesotrione after the geometry optimization followed by the complete vibrational analysis. These data serve for evaluating the adiabatic ionization energy *E*_i_ and adiabatic electron affinity *E*_eg_; these allow determining the derived electronic properties such as electronegativity $\chi$, hardness *η,* and electrophilicity *ω*. The reaction Gibbs energies serve for determining the absolute oxidation and reduction potentials at standard conditions.

**Table 2:** Electronic energy and Gibbs energy for nitisinone, mesotrione, and their ions in water^a^

Molecule/ion	L^+^	L^0^	L^−^
Nitisinone–M1: B3LYP/def2-TZVPD			
* E*^el^(L^*q*^) in optimized str of L^*q*^	− 796,595.18	− 796,759.13	− 796,844.30
* G*^ø^(L^*q*^) in optimized str of L^*q*^	− 796,484.23	− 796,646.02	− 796,733.73
* G*^ø^(L^*q*^)–*E*^el^(L^*q*^)	110.95	113.11	110.57
Mesotrione–M1: B3LYP/def2-TZVPD			
* E*^el^(L^*q*^) in optimized str of L^*q*^	− 953,990.47	− 954,154.48	− 954,241.02
* G*^ø^(L^*q*^) in optimized str of L^*q*^	− 953,858.94	− 954,021.46	− 954,109.63
* G*^ø^(L^*q*^)–*E*^el^(L^*q*^)	131.53	133.02	131.39
Nitisinone–M2: DLPNO-CCSD(T)/aug-cc-pVTZ and aug-cc-pVTZ/C			
* E*^el^(L^*q*^) in fixed str of L^*q*^	− 795,768.51	− 795,966.92	− 796,049.93
Mesotrione–M2: DLPNO-CCSD(T)/aug-cc-pVTZ and aug-cc-pVTZ/C			
* E*^el^(L^*q*^) in fixed str of L^*q*^	− 952,979.81	− 953,201.91	− 953,285.61

^a^Energy units kcal mol^−1^

According to Table 3, the electronic redox properties (*E*_i_, *E*_eg_, *χ*, *η*, *ω*) and thermodynamic properties (*E*_ox_^ø^, *E*_red_^ø^) of nitisinone are essentially the same as for mesotrione. The chemical hardness is a measure of the willingness of the molecule against the electron transfer; for nitisinone and mesotrione, it is *η* = 39 kcal mol^−1^ (rather low value). The electrophilicity index is extremely high: *w* = 200 kcal mol^−1^. Consequently, the absolute reduction potential is very high *E*_red_^ø^ =  + 3.80 V; for amino acids and small biogenic molecules, it is typically ~  + 1 V when the same methodology (B3LYP) is used. Noticeably, *ω* is defined on the basis of electronic energies of L^0^, L^+^, and L^−^ species and it is common for oxidation and reduction processes. On the contrary, redox potential is derived from the standard reaction Gibbs energies and is different for oxidation and reduction processes.

**Table 3:** Molecular descriptors calculated by DFT-B3LYP/def2-TZVPD (M1) and DLPNO-CCSD(T)/aug-cc-pVTZ (M2) method in water using adiabatic ionization/affinity processes^a^

Method	Molecule	*E*_i_	*E*_eg_	*χ*	*η*	*ω*	*E*_ox_^ø^	*E*_red_^ø^	*p*	*Q*	*α*	*E*_zpe_	*S·T*^ø^
M1	Nitisinone	164	− 86	125	39	200	− 7.05	3.80	7.345	− 100.1	270.4	142.7	42.7
M2	Nitisinone	198	− 83	140	57	173	− 8.59 ^b^	3.60 ^b^	7.815	− 101.6	-	-	-
M1	Mesotrione	164	− 86	125	39	200	− 7.02	3.82	8.868	− 110.2	309.5	163.5	44.4
M2	Mesotrione	222	− 84	153	69	170	− 9.63 ^b^	3.64 ^b^	9.516	− 111.9	-	-	-

^a^Energy units kcal mol^−1^; *E*_o_^ø^ and *E*_r_^ø^/V; *p*/debye, *Q*/e*a*_0_^2^, *α*/*a*_0_^3^

^b^Approximate expression *E*_r_^*^ =  − Δ_r_*E*^el^/*F*, *F* = 96,485 A s⋅mol^−1^

An inspection to Table 3 confirms that the overall temperature-dependent correction to Gibbs energy *G*^ø^(L^*q*^) − *E*^el^(L^*q*^) is about 111 and 133 kcal mol^−1^ which in fact is only 0.01% of the total energy. Therefore, it might be possible to express the redox potential by an approximate formula *E*_r_^*^ =  − Δ_r_*E*^el^/*F* when the Gibbs energy is not available due to a very tedious numerical evaluation of the vibrational frequencies. The ionization energy and/or the electron affinity then serve for the reaction electronic energies.

Some of the molecular properties of nitisinone and mesotrione are classified as bulky properties and they increase with increasing molar mass: absolute value of the quadrupole moment |*Q*|, isotropic dipole polarizability *α*, zero-point energy *E*_zpe_, and the total entropic term *S·T*^ø^ belong to them. The dipole moment reflects a delicate separation of the barycenters of positive–negative charges: *p* = 6.3 and 8.9 D for nitisinone and mesotrione, respectively.

Application of a more exact DLPNO-CCSD(T) method (M2) to above-mentioned neutral and ionized species in geometries optimized by B3LYP method (M1) brings results presented in Table 3. The ionization energy is high (*E*_i_ = 198 and 222 kcal mol^−1^, higher than by M1), and the electron affinity is very negative (about *E*_eg_ ~  − 83 kcal mol^−1^, similar to M1). High ionization energy is reflected into increased molecular electronegativity *χ* and increased chemical hardness *η*. The resulting electrophilicity index is very high (*ω* ~ 170 kcal mol^−1^) which predetermines high reduction potential.

**Fig. 2:**
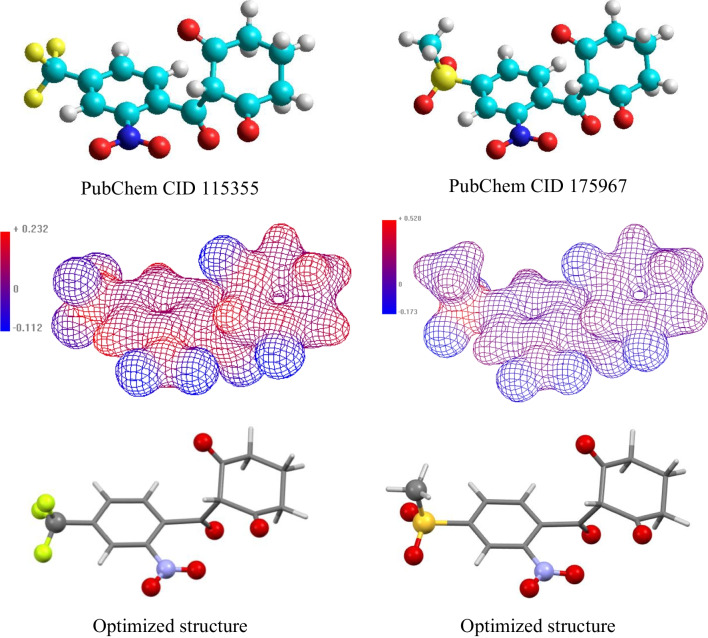
The optimized molecular structure (by B3LYP) and 3D mapped isosurface of the molecular electrostatic potential (by AM1 method); contours 0.03 *ea*_0_^−1^, *a*_0_ *bohr* unit. Color scale: blue, negative; red, positive. Left, nitisinone; right, mesotrione

The electrostatic potential distributed along the surface of the molecule is taken against a unit charge and is known as the molecular electrostatic potential (MEP) [23, 24]. It is visualized as a three-dimensional contour map plotted on the isovalue surface of charge density. It identifies sites possessing positive or negative values that are suitable for nucleophilic and/or electrophilic interactions along the molecule. HyperChem software has been exploited for generating MEP [25] that is drawn in Fig. 2 along with the optimized molecular structures. With expectations, high negative potential occurs in the sites of electronegative atoms such as fluorine and oxygen (blue colored); positive potential is delocalized over the rings and localized at the sulfur atom and/or carbon od the –CF_3_ moiety (red colored).

**Fig. 3:**
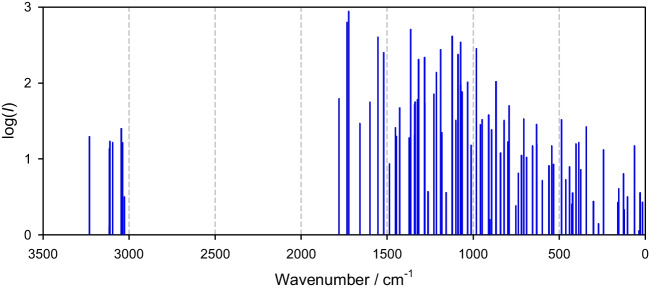
Calculated IR spectrum for nitisinone in water (102 transitions)

**Fig. 4:**
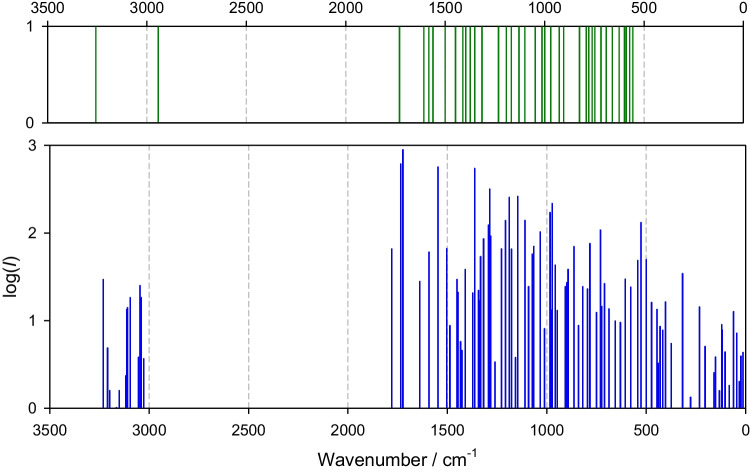
Calculated IR spectrum (bottom panel, in water, 93 transitions) in comparison with reported significant transitions for solid-state mesotrione (top panel, data collected above 500 cm^−1^ [20])

The calculated IR spectra over the whole wavenumber range are shown in Fig. 3 for nitisinone and in Fig. 4 for mesotrione. The DFT-B3LYP is not a perfect tool for reproducing the vibrational spectra quantitatively; however, after some scaling (scaling factor about 0.96) this method yields acceptable results [26]. Both spectra can be divided into three domains: above 3000 (I), below 1700 (II), and below 250 (III) cm^−1^. The experimental window is 500–4000 cm^−1^. For nitisinone, the high-frequency domain is limited by medium-intensity bands at I, 2950–3250 cm^−1^ where a number of low-intensity transitions exist in between. This feature is well reproduced by calculations. There is a broad silent gap between domains I and II. Domain II starts with high-intensity peaks at 1700 cm^−1^ followed by a huge number of resolved, weakly resolved, or oddly resolved transitions [20]. For the far-IR domain III, experimental data are missing.

Last but not least, some notes regarding biological activity are given. Both studied compounds, nitisinone and mesotrione, are potent inhibitors of 4-hydroxyphenylpyruvate dioxygenase (HPPD), 40–50 kDa. In active sites, this enzyme contains Fe^II^ in hexacoordinate form [27, 28]. In plants, HPPD exists in its monomeric form but dimers or tetramers were identified in bacteria. In plants, HPPD transforms tyrosine into other products necessary for living: after several steps, the lack of tyrosine prevents the formation of chlorophyll, which in turn initiates plant death. HPPD inhibitors include not only nitisinone and mesotrione but also certain related species such as triketones sulcotrione and NTBC (2-(2-nitro-4-trifluoromethylbenzoyl)-1,3-cyclohexanedione). It could be hypothesized that a direct oxidation of Fe^II^ to Fe^III^ by strong oxidizing agents (mesotrione and nitisinone) may destroy the functionality of HPPD. However, experiments show that the {HPPD·Fe^II·^NTBC} adduct under aerobic conditions maintains typical electronic transition at 450 nm for a long time, which confirms the presence of Fe^II^ [29]. There is an important difference in price of nitisinone and mesotrione (at the moment mesotrione is 15 times cheaper).

## Conclusions

In conclusion, DFT-B3LYP calculations show that two derivatives of β-triketones, nitisinone and mesotrione, possess essentially molecular parameters in water such as energies of HOMO and LUMO, the ionization energy and electron affinity, and derived electronic properties such as molecular electronegativity, chemical hardness, and electrophilicity index. They differ in the dipole moment (high value *p* = 7.3 and 8.9 debye) and bulk properties such as isotropic polarizability (*α* = 270 and 309 *a*_0_^3^), solvated surface and volume, zero-point vibration energy (143 and 163 kcal mol^−1^), and the total entropic contribution (*S·T*^ø^ = 42 and 44 kcal mol^−1^). The value of the absolute reduction potential is very high (*E*_red_^ø^ = 3.80 and 3.82 V) so that these species are effective oxidizing agents. DLPNO-CCSD(T) method, that includes explicitly the correlation energy, applied to nitisinone and mesotrione confirms qualitative predictions of more approximate B3LYP in water: high adiabatic ionization energy, very negative electron affinity (*E*_eg_ ~  − 83 kcal mol^−1^), high electronegativity, low chemical hardness, high electrophilicity index (*ω* = 170 kcal mol^−1^), and high dipole moment (*p* = 7.8 and 9.5 debye).

## Data Availability

Output protocols of calculations are available on request to the corresponding author.
